# Identification of biomarkers associated with immune scores in diabetic retinopathy

**DOI:** 10.3389/fendo.2023.1228843

**Published:** 2023-10-05

**Authors:** Yi Zhang, Weidong Zhu, Jianming Wang, Yi Zuo

**Affiliations:** ^1^ Department of Ophthalmology, The Second Affiliated Hospital of Xi’an Jiaotong University, Xi’an, China; ^2^ Department of Spinal Surgery, No. 215 Hospital of Shaanxi Nuclear Industry, Xianyang, China; ^3^ Department of Neurosurgery, No. 215 Hospital of Shaanxi Nuclear Industry, Xianyang, China

**Keywords:** diabetic retinopathy, immune-associated genes, bioinformatics, immune, biomarkers

## Abstract

**Background:**

Diabetic retinopathy (DR) causes irreversible visual impairment in diabetes mellitus (DM) patients. Immunity played a crucial role in DR. Nevertheless, the triggering mechanism of DR was not yet thorough enough. Herein, we aim to identify the immune-associated genes as biomarkers associated with immune scores that can distinguish early DR from DM without DR.

**Methods:**

In this study, total RNA of peripheral blood mononuclear cell (PBMC) samples from 15 non-proliferative DR patients and 15 DM patients without DR were collected and the transcriptome sequencing data were extracted. Firstly, the target genes were obtained by intersecting the differentially expressed genes (DEGs), which were screened by “limma”, and the module genes (related to immune scores), which were screened by “WGCNA”. In order to screen for the crucial genes, three machine learning algorithms were implemented, and a receiver operating characteristic (ROC) curve was used to obtain the diagnostic genes. Moreover, the gene set enrichment analysis (GSEA) was performed to understand the function of diagnostic genes, and analysis of the proportions of immune cells and their association with diagnostic genes was performed to analyze the pathogenesis of DR. Furthermore, the regulatory network of TF–mRNA–miRNA was built to reveal the possible regulation of diagnostic genes. Finally, the quantitative real-time polymerase chain reaction (qRT-PCR) was performed to verify the mRNA level of diagnostic genes.

**Results:**

A total of three immune-associated diagnostic genes, namely, *FAM209B*, *POM121L1P*, and *PTGES*, were obtained, and their expression was increased in PBMC samples of DR, and qRT-PCR results confirmed these results. Moreover, the functions of these genes were associated with immune response. The expression of *POM121L1P* and *PTGES* was significantly negatively associated with naive B cells, and the expression of *FAM209B* was significantly negatively associated with immature dendritic cells. Moreover, *ESR1* could regulate both *FAM209B* and *PTGES*.

**Conclusion:**

This study identified three immune-associated diagnostic genes, *FAM209B*, *POM121L1P*, and *PTGES*, as biomarkers associated with immune scores in DR for the first time. This finding might proffer a novel perspective of the triggering mechanism of DR, and help to understand the role of immune-associated genes in the molecular mechanism of DR more deeply.

## 1 Introduction

Among the ocular complications of diabetes mellitus (DM), the incidence of diabetic retinopathy (DR) is high, reaching 34.6% in the total population of DM patients. More seriously, the incidence of DR-related blindness was 2.6% (1). As of 2020, there were approximately 103 million DR patients around the world, and the number of DR patients may increase to 160 million by 2045 (2). DR causes irreversible visual impairment in DM patients, and is the main cause of blindness in the adult working population worldwide (2), which has brought heavy economic pressure (3). However, the current medical methods can neither completely prevent nor cure DR. It has been found that the emergence and progression of DR were not only related to the duration of hyperglycemic state and the level of blood glucose control (4), but also related to the different genetic susceptibility of different individuals and abnormal immune responses (5). Thus, it is crucial to continuously search for the pathogenesis involved in DR, especially the triggering mechanism of early DR from a new perspective and develop new prevention and treatment methods accordingly.

Relevant studies have, to some extent, revealed that immunity played a pivotal role during the occurrence and progression of DR (6, 7). Numerous immune cells may play potential roles in DR. In terms of retinal innate immunity, it is found that microglia are activated at the early stage of DR, and then release proinflammatory mediators and attract more immunocytes (8). In terms of adaptive immunity, T lymphocyte infiltration promotes the secretion of inflammatory cytokines as well. Furthermore, cellular immune response and phagocytic cell-dependent inflammatory effect are initiated, thus participating in the pathological process of DR (9). It has also been found that the densities of B lymphocytes significantly increased in the fibrovascular membranes of active proliferative diabetic retinopathy (PDR) patients (10). However, biomarkers associated with immune scores that can distinguish early DR patients from DM patients without DR are still not thorough enough.

Owing to the widespread application of RNA-sequencing, it has become more convenient to find new therapeutic targets for DR. Therefore, the differentially expressed immune-associated genes between the peripheral blood mononuclear cell (PBMC) samples of DR and DM patients were screened for in-depth analyses. The immune-associated diagnostic genes that could form the diagnostic model of DR were confirmed through machine learning algorithms and were then verified in subsequent experiments. Furthermore, the functions of diagnostic genes, the proportions of immune cells, and their association with diagnostic genes were analyzed to reveal the possible pathogenesis mechanism of DR occurrence. Furthermore, the up-/downstream regulatory mechanisms of diagnostic genes were predicted to further reveal the potential regulation of these immune-associated diagnostic genes in DR.

## 2 Materials and methods

### 2.1 Data collection, RNA extraction, and library construction

In this study, 456 immune-related genes were obtained from the ImmPort database (https://www.immport.org/home) (**Supplementary Table 1**), and PBMC samples from the total of non-proliferative DR (*n* = 15) and DM without DR (*n* = 15) patients were acquired for RNA extraction. Patients with type 1 DM, gestational DM, special type DM, hypertension, coronary heart disease, chronic obstructive pulmonary disease, malignant tumor, stroke, glaucoma, uveitis, and other retinal diseases are excluded. This study was approved by the Second Affiliated Hospital of Xi’an Jiaotong University ethical review committee (approval number: 2023253).

Total RNA was isolated and purified using TRIzol reagent (Invitrogen, Carlsbad, CA, USA), and the RNA amount and purity of each sample were quantified using NanoDrop ND-1000 (NanoDrop, Wilmington, DE, USA). The poly(A) RNA was fragmented into small pieces and reverse-transcribed to create the cDNA. Next, the ligated products were amplified with polymerase chain reaction (PCR). Last, we performed the 2 × 150- bp paired-end sequencing (PE150) on an Illumina NovaseqTM 6000 (LC-Bio Technology CO., Ltd., Hangzhou, China). Finally, the mRNA sequencing data of 15 DR and 15 DM samples were obtained. Based on it, the sequencing data were analyzed by “FastQC” (version 0.11.9), and the low-quality data were filtered to remove contamination and adaptor sequences and to obtain the clean data finally. Furthermore, the clean data were aligned to the reference genome (GRCh37) by “hisat2” (version2.2.1).

### 2.2 Identification of target genes of DR

The differentially expressed genes (DEGs) between DR (*n* = 15) and DM (*n* = 15) samples were compared by “limma” R package (version 3.48.3) (|log2FC| > 1, *p* < 0.05) (11). The function analysis of DEGs was conducted by the “clusterprofiler” R package (version 4.0.2) (adj. *p* < 0.05, count ≥ 1) (12). On the other hand, the immune scores of all samples (*n* = 30) were calculated based on the expression profiles of 456 immune-related genes from the ImmPort database by the “GSVA” R package (version 1.44.5) (13). The co-expression network was constructed by the “WGCNA” R package (version 1.70-3), and the immune scores were utilized as the trait to screen relevant module genes (14). Then, the target genes were obtained by intersecting the module genes and DEGs using “venn”.

### 2.3 Construction of the diagnostic model of DR

Firstly, three methods, namely, the “Boruta” method (15), the least absolute shrinkage and selection operator (LASSO) analysis (16), and the support vector machine recursive feature elimination (SVM-RFE) method, were utilized for screening the characteristic genes, respectively. Then, the key genes were obtained by crossing three sets of genes. Thirdly, the receiver operating characteristic (ROC) curves of each key gene and the whole genes were drawn to study the ability of key genes to distinguish DR from the DM population. The genes which the area under the ROC curve (AUC) value greater than 0.7 were defined as the diagnostic genes. Moreover, the expressions of the diagnostic genes between DR (*n* = 15) and DM (*n* = 15) were compared by the “rank-sum test”.

Based on it, the nomogram model with these diagnostic genes was constructed by the “rms” R package (version 6.1-0). Then, the calibration curve, ROC curve, and decision curve analysis (DCA) were drawn to verify the validity of the nomogram.

### 2.4 The functions of diagnostic genes and landscape of immune cells in PBMC analyses

On the one hand, the gene set enrichment analysis (GSEA) was utilized for studying the pathways of each diagnostic gene by the “clusterProfiler” R package (version 4.4.4) (|NES| > 1, NOM *p* < 0.05, *q* < 0.25), respectively (11).

On the other hand, the proportions of immune cells between DR (*n* = 15) and DM (*n* = 15) PBMC samples were calculated by the “xCell” algorithm and compared by the “rank-sum test”. Moreover, the correlations between diagnostic genes and differential immune cells, as well as between diagnostic genes and marker genes of differential immune cell, were further studied by “Spearman”.

### 2.5 Molecular mechanism analyses

The target transcription factors (TFs) and the target miRNAs of diagnostic genes were predicted in Cistrome database (RP score > 0.3) and miRwalk database (energy ≤ −30), respectively. Then, the TF–mRNA–miRNA network was constructed by “Cytoscape” (17).

### 2.6 Validation of the expression of diagnostic genes

Quantitative real-time PCR (qRT-PCR) was performed to validate the expression of diagnostic genes in DR (*n* = 10) and DM (*n* = 10) PBMC samples. Total RNA was extracted and the qPCR reactions were performed using the SureScript First-strand cDNA synthesis kit (Servicebio, Wuhan, China). The forward and reverse primers were as shown in **Supplementary Table 2**. The relative gene expression was presented by the comparative CT method.

### 2.7 Statistical analysis

All analyses were conducted using R language. Experimental data were statistically analyzed by GraphPad Prism (version 5) software. Student’s *t*-test was used for the comparison of the DR and DM groups. If not specified above, *p* < 0.05 was considered as statistically significant.

## 3 Results

### 3.1 Data quality control and pre-processing

As shown in **Supplementary Table 3**, the base calling error of all sequencing fragments was less than 1/1,000 (QC% > 30), the depth was greater than 100× (total reads > 10 M), and the comparison ratio of 29 samples was higher than 90%. These results showed that the sequencing results could meet the needs of subsequent analyses.

### 3.2 A total of 15 immune-associated target genes were obtained in DR

**Figure 1:**
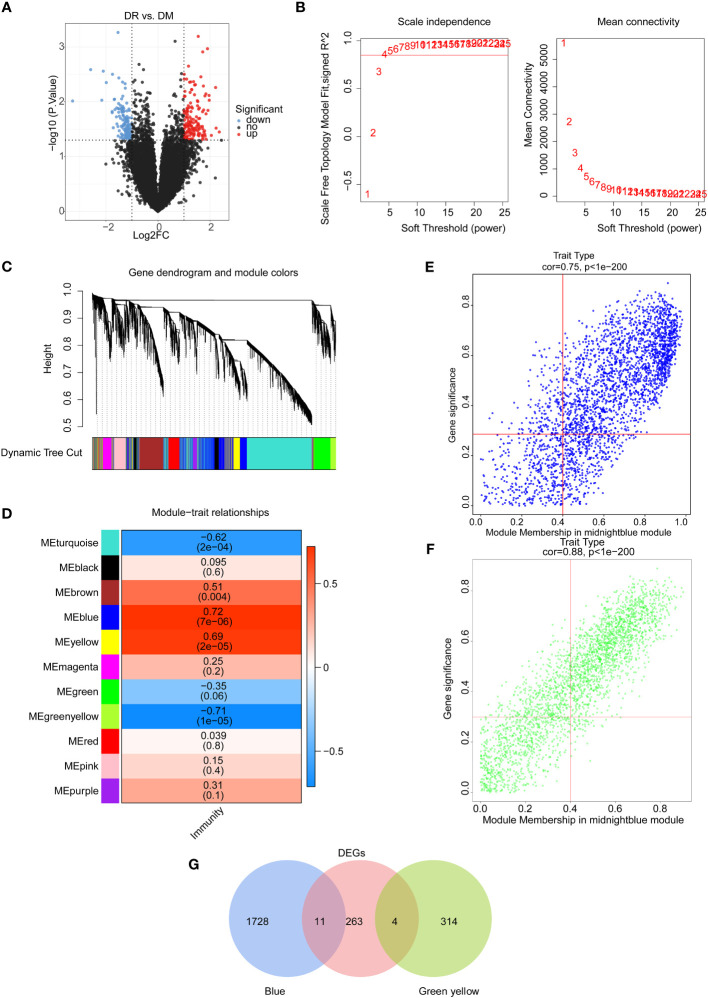
Identification of immune-associated differential expressed genes between the DR group and the DM group. **(A)** Volcano map of differentially expressed genes between the DR group and the DM group. **(B)** Selection of soft threshold value. **(C)** Hierarchical clustering tree diagram of genes, where genes clustered to the same branch are divided into the same module, and different colors represent different modules. **(D)** Heat map of the relationship between gene modules and traits using immune scores as phenotypes. Scatter plots of module membership and gene significance for key module genes, including **(E)** the blue module and **(F)** the green–yellow module. **(G)** Venn diagram of candidate differentially expressed genes.

In order to screen the potential immune-associated target genes in DR, the differentially expressed analysis was first employed for comparing and analyzing gene expression patterns between DR and DM PBMC samples. There were 278 DEGs (146 upregulated and 132 downregulated) between 15 DR and 15 DM samples (**Figure 1A**; **Figure S1A**). In the perspective of function, these 278 DEGs were enriched to 209 Gene Ontology (GO) functions, including Wnt signaling pathway and digestive tract morphogenesis. It was worth noting that 11 target genes were involved in the regulation of GTPase activity. Furthermore, these genes were enriched to 13 Kyoto Encyclopedia of Genes and Genomes (KEGG) pathways, including regulation of lipolysis in adipocytes, Wnt, and Hedgehog signaling pathways (**Figures S1B, C**, **Supplementary Tables 4**, **5**).

On the other hand, the immune scores of all samples were calculated on the basis of the expression profiles of 456 immune-related genes from the ImmPort database, and the weighted gene co-expression network analysis (WGCNA) was conducted to select the key module genes associated with immune score. Sample clustering analysis was first implemented and the results showed that there were no outlier samples (**Figure S2A**). Then, the optimal soft threshold value was identified as five to ensure the scale-free distribution of the network, and a total of 11 modules were obtained based on the genes with the similar expression patterns (**Figures 1B, C**, **S2B**). Among them, the blue module had a significantly positive correlation with the immune score (*R* ^2 ^= 0.72, *p* = 7e-6), and the green–yellow module had a significantly negative correlation with the immune score (*R* ^2^ = −0.71, *p* = 1e-6) (**Figure 1D**). Hence, 2,057 genes in these two modules with |GS| > 0.3 and |MM| > 0.4 were screened as key module genes related to immune scores for subsequent analysis (**Figures 1E, F**).

Furthermore, by intersecting 2,057 immune score-related module genes and 278 DEGs between DR and DM samples, a total of 15 common genes, namely, *CCDC144B*, *CFAP298-TCP10L*, Family with sequence similarity 209 member B (*FAM209B*), *GUSBP17*, *IGKJ3*, *KANTR*, *KRT1*, *MEF2C-AS2*, *OCIAD1-AS1*, POM121 membrane glycoprotein-like 1 pseudogene (*POM121L1P*), Prostaglandin E synthetase (*PTGES*), *TRGV5P*, *VWCE*, *WNT4*, and *ZNF876P*, were obtained as significantly differential target genes relevant to immune scores (**Figure 1G**).

### 3.3 Three diagnostic genes were used to construct the diagnostic model of DR

**Figure 2:**
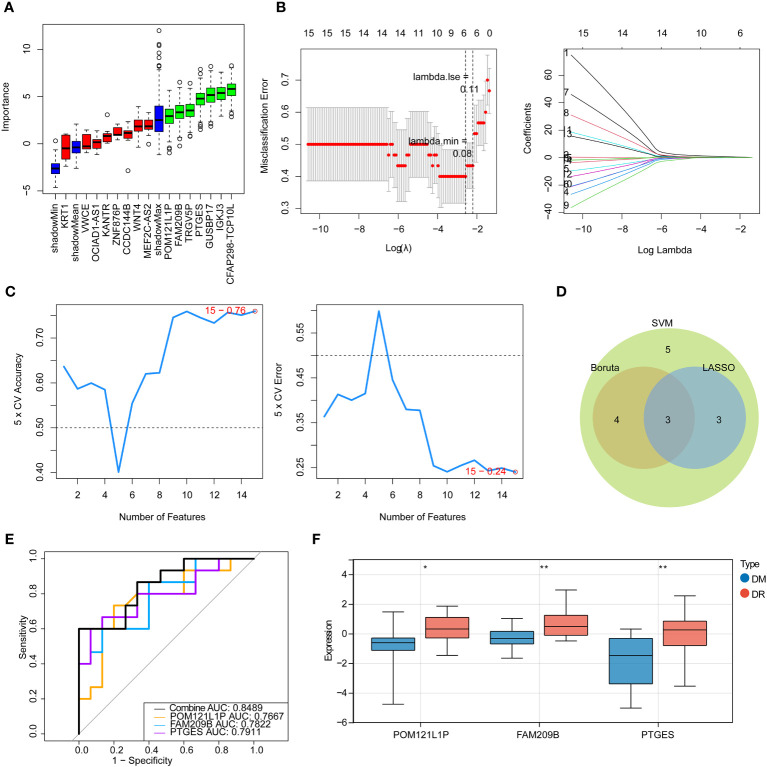
Identification of immune-associated diagnostic genes for DR. **(A)** Boruta analysis for identifying feature genes. The red, yellow, and green box charts represent the rejected, tentative, and confirmed *Z* score attributes, respectively. We select green as the important feature gene. **(B)** Screening for characteristic genes by LASSO regression analysis. **(C)** Screening for characteristic genes by SVM-RFE analysis. **(D)** Venn diagram of genes intersected by results of Boruta, LASSO, and SNM analysis. **(E)** ROC curve of key genes. **(F)** Expression of key diagnostic genes in the DR group and the DM group. *p < 0.05, **p < 0.01.

Next, three machine learning methods, namely Boruta (**Figure 2A**), LASSO (**Figure 2B**), and SVM-RFE (**Figure 2C**), were utilized to screen the key diagnostic genes based on the target genes as mentioned above, where seven candidate feature genes, namely, *CFAP298-TCP10L*, *FAM209B*, *GUSBP17*, *IGKJ3*, *POM121L1P*, *PTGES*, and *TRGV5P*, were screened by Boruta analysis; six characteristic genes, namely, *FAM209B*, *KRT1*, *POM121L1P*, *PTGES*, *VWCE*, and *WNT4*, were screened by LASSO analysis; and all target genes were defined as the feature genes by SVM-RFE analysis. Furthermore, three key genes shared by three diagnostic models, namely, *FAM209B*, *POM121L1P*, and *PTGES*, were obtained by crossing the above three sets of genes (**Figure 2D**).

ROC analysis was used to determine the predictive performance of three key genes as well as the gene-based diagnostic models to distinguish DR from DM samples. It can be seen that the AUC values of each key gene were greater than 0.7, and when all key genes were considered as a whole, the AUC value was 0.8489 (**Figure 2E**), suggesting that *FAM209B*, *POM121L1P*, and *PTGES* could be defined as good diagnostic genes for subsequent analyses. Moreover, the expression profiles of all diagnostic genes between DR and DM samples were extracted in the sequencing results, and it can be seen that these genes are significantly highly expressed in the DR group (**Figure 2F**).

**Figure 3:**
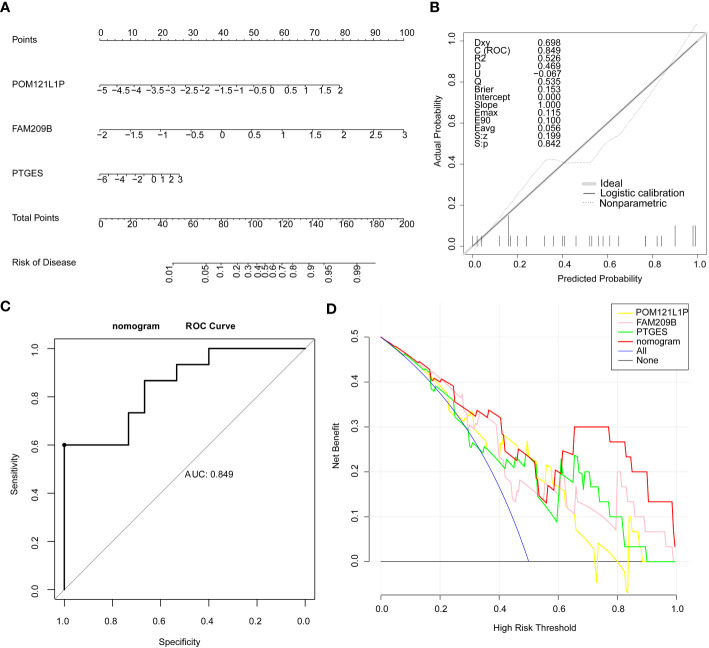
Nomogram on the basis of three diagnostic genes was constructed for clinical utilize. **(A)** Nomogram. **(B)** Calibration curve of the nomogram. **(C)** ROC curve of the nomogram. **(D)** DCA curve of the nomogram.

Furthermore, the nomogram with three diagnostic genes was constructed for clinical use. The calibration curve results showed that the slope closed to 1, indicating that the nomogram had an accurate ability for predicting the risk of DR (**Figures 3A, B**). The AUC value of the nomogram was 0.849, and the results of the DCA further suggested that the benefit rate of the nomogram model was higher than each individual gene (**Figures 3C, D**). All of these results indicated that the nomogram, by converting the expression of three key genes into a total score, could be taken into consideration for clinical use as well.

### 3.4 The functions of diagnostic genes were associated with various immune responses

**Figure 4:**
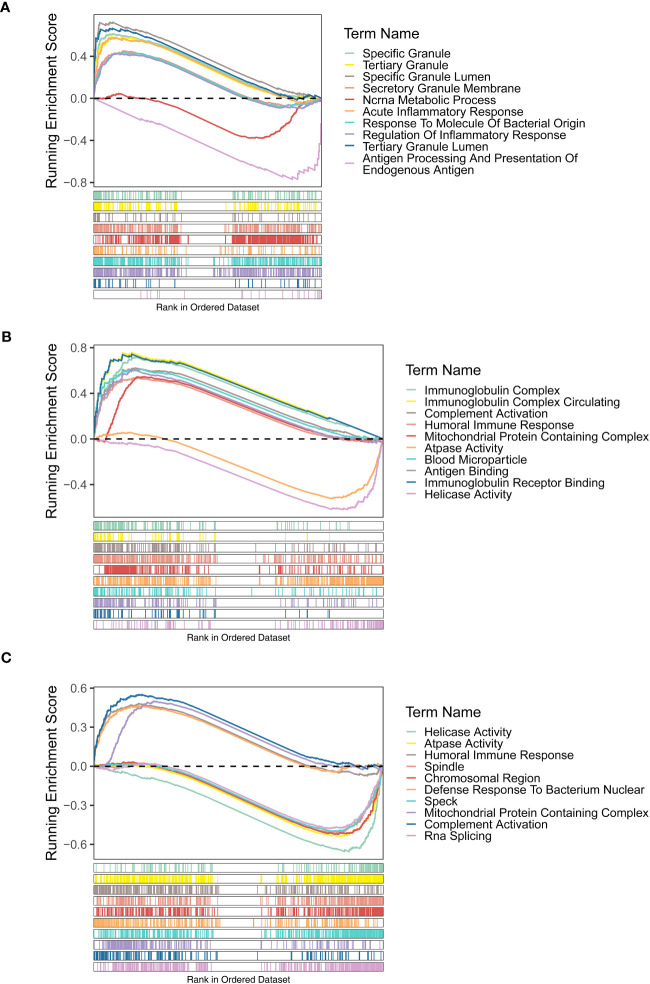
Gene set enrichment analysis (GSEA) results (GO terms) of three diagnostic genes *FAM209B* **(A)**, *POM121L1P* **(B)**, and *PTGES* **(C)**.

The GSEA results of three diagnostic genes revealed that the biological process (BP) of regulation of inflammatory response, acute inflammatory response, acute phase response, etc., were highly enriched by *FAM209B*. It is worth noting that *POM121L1P* and *PTGES* were related to many same functions, including humoral immune response, immunoglobulin complex, mitochondrial protein containing complex, and ATPase activity. In addition, the functions of antigen binding and immunoglobulin receptor binding were also highly enriched by *POM121L1P* (**Figures 4A–C**).

**Figure 5:**
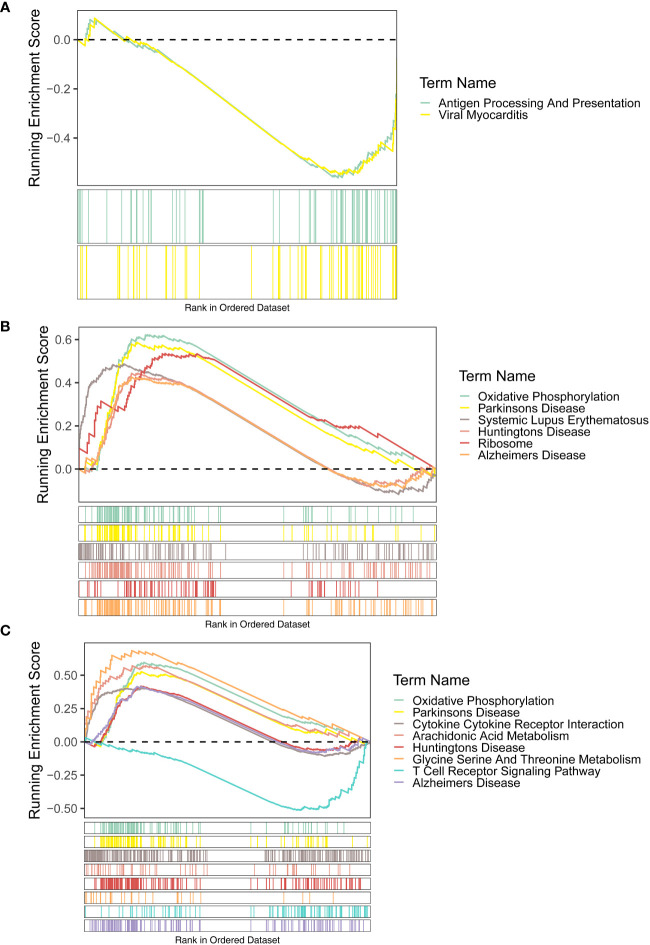
GSEA results (KEGG terms) of three diagnostic genes *FAM209B* **(A)**, *POM121L1P* **(B)**, and *PTGES* **(C)**.

For the KEGG enrichment results, the signaling pathways of antigen processing and presentation and viral myocarditis were highly enriched by *FAM209B*. *POM121L1P* and *PTGES* were common to the pathways of Alzheimer’s disease, Huntington’s disease, Parkinson’s disease, and oxidative phosphorylation (**Figures 5A–C**).

### 3.5 The correlations of diagnostic genes and immune cells

**Figure 6:**
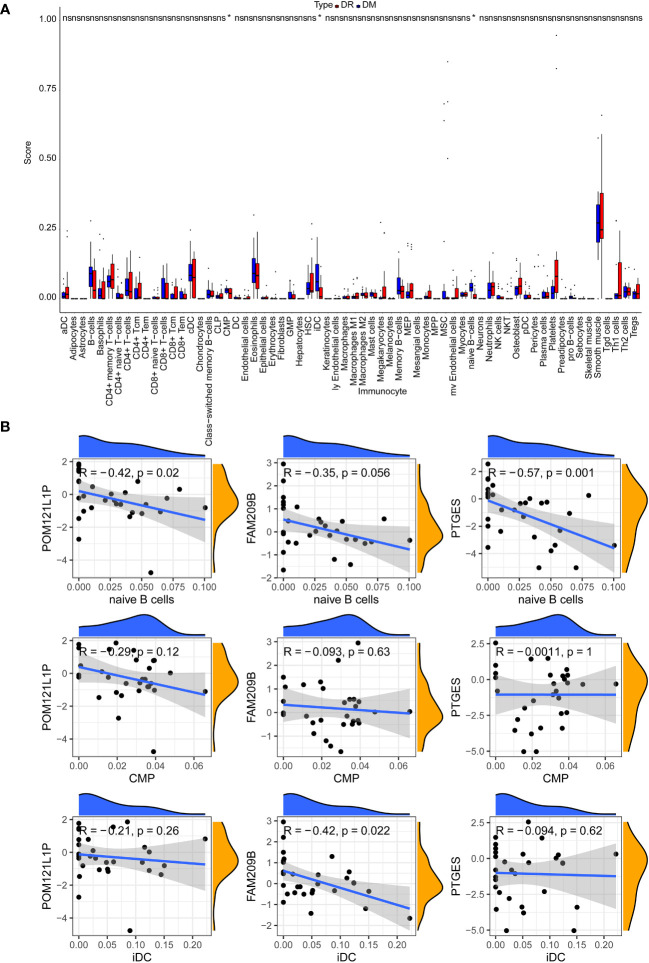
The analysis of circulating immune cells in PBMCs of the DR group and the DM group. **(A)** Differences in the proportion of circulating immune cells in PBMCs between the DR group and the DM group. **(B)** Scatter chart of the correlation between key genes and differential immune cells. *p < 0.05; ns, no significance.

**Figure 7:**
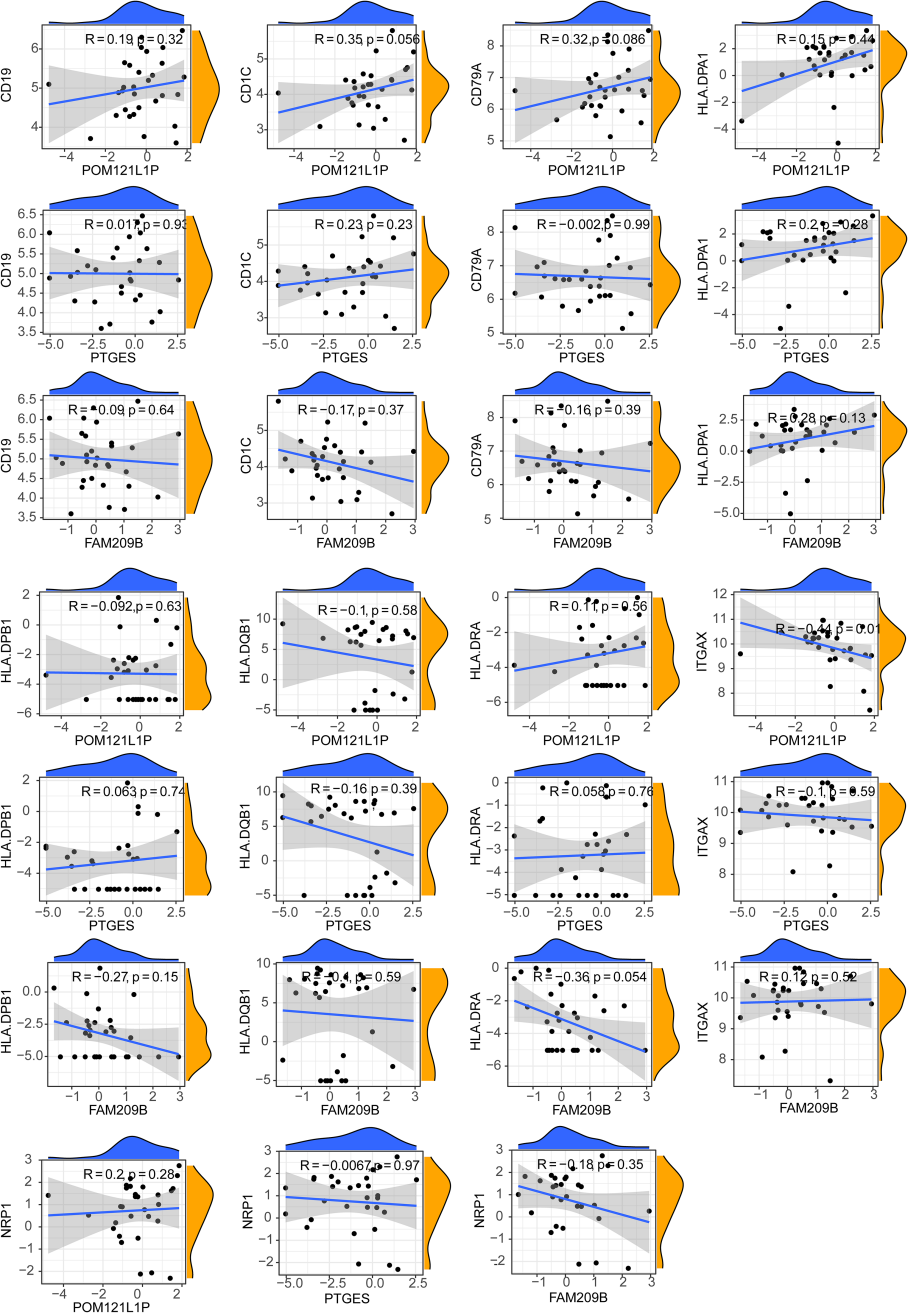
Scatter chart of the correlation between key genes and differential cell marker genes.

After calculating the immune cell proportions of each PBMC sample using xCell tools, a total of three immune cells, namely, common myeloid progenitor cell (CMP), immature dendritic cell (iDC), and naive B cell, were found to be significantly decreased in the DR group (**Figure 6A**). Among them, *POM121L1P* and *PTGES* were significantly negatively associated with naive B cell, and *FAM209B* was significantly negatively associated with iDC (*p* < 0.05) (**Figure 6B**). In addition, there was a significantly strong negative correlation between *POM121L1P* and *ITGAX* (the marker gene of iDC) (*p* = 0.015, |cor| = 0.44) (**Figure 7**).

### 3.6 Molecular mechanism analyses of diagnostic genes

**Figure 8:**
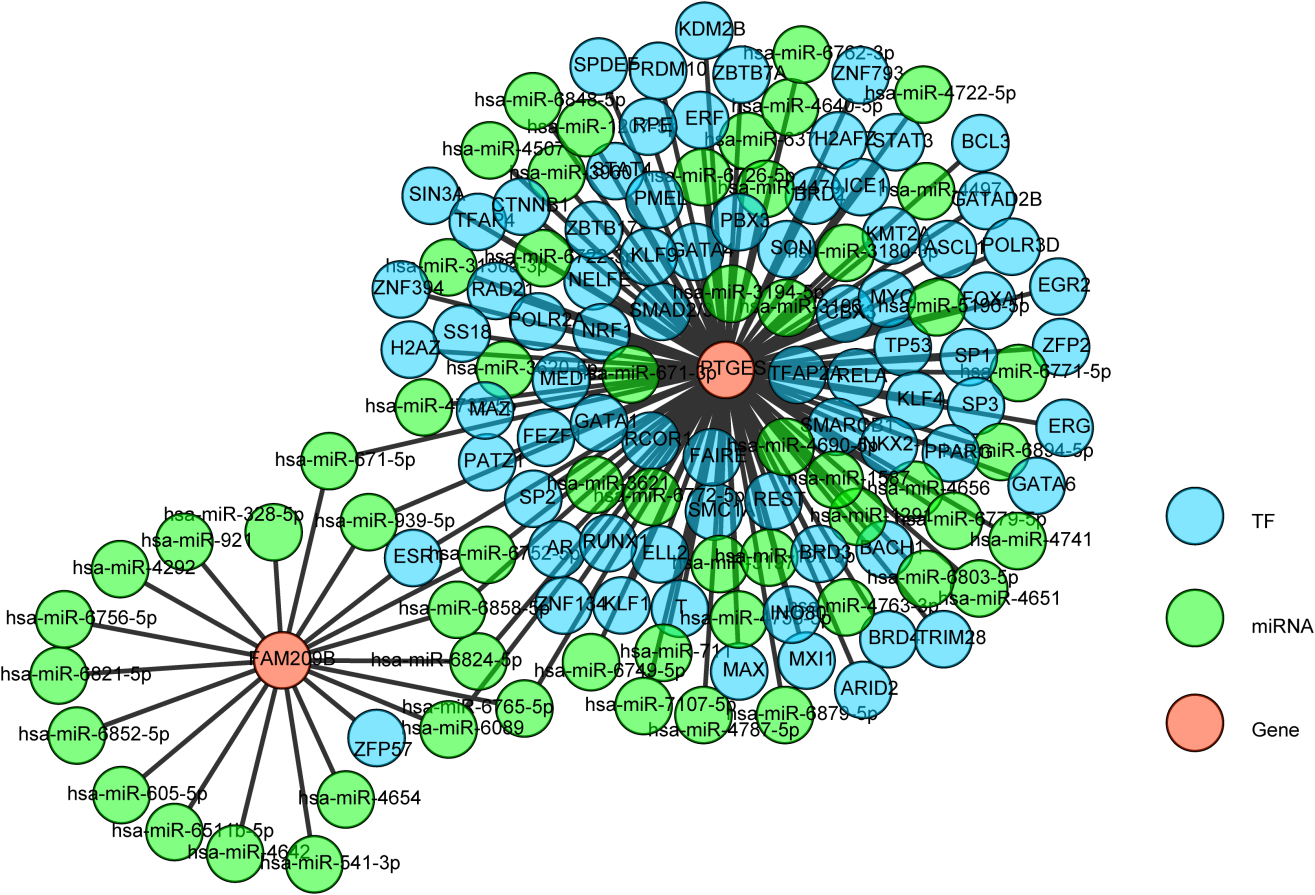
The TF-miRNA-mRNA network of key genes.

For the potential regulatory network of diagnostic genes, the TF–mRNA–miRNA network contained 59 miRNAs, 76 TFs, and 2 diagnostic genes. Among them, *FAM209B* had 18 targeted miRNAs and *PTGES* had 48 targeted miRNAs. Notably, there were seven common miRNAs (hsa-miR-671-5p, hsa-miR-939-5p, hsa-miR-6752-5p, hsa-miR-6858-5p, hsa-miR-6824-5p, hsa-miR-6765-5p, and hsa-miR-6089) between *FAM209B* and *PTGES*. On the other hand, *FAM209B* had two targeted TFs and *PTGES* had 75 targeted TFs, and it was worth noting that the estrogen receptor 1 (*ESR1*) could regulate both *FAM209B* and *PTGES* at the same time (**Figure 8**).

### 3.7 Expression verification of immune-associated diagnostic genes

**Figure 9:**
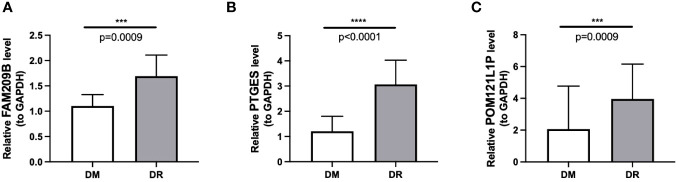
Differential mRNA expression of three immune-associated diagnostic genes FAM209B **(A)**, PTGES **(B)**, and POM121L1P **(C)** between the DR group and the DM group by qRT-PCR. ***p < 0.001, ****p < 0.0001.

The qRT-PCR results also showed that the expressions of the three diagnostic genes were significantly higher in DR PBMC samples (*n* = 10) than in DM PBMC samples (*n* = 10), which were in accordance with the sequencing results (*p* < 0.001) (**Figure 9**).

## 4 Discussion

Currently well-known risk factors such as age, disease duration, and hemoglobin A1c are not effective in identifying patients with early DR (18), and the differences between individuals might be related to the interaction of multiple pathophysiological factors, among which the influence of immune-mediated inflammatory response is particularly critical (19), but until now, the immune-related mechanisms contributing to the onset of DR have not been thoroughly elucidated. DR often goes through a long false silent phase before diagnosis (20), and frequent and regular fundus examination is an effective method for detecting the occurrence of DR. However, on the one hand, many DM patients cannot complete fundus examination regularly before visual impairment occurs; on the other hand, many DM patients are complicated with cataracts at the same time, which hinders the clear imaging of the fundus (21). Therefore, a new diagnostic method for early DR needs to be explored. Compared with aqueous humor or vitreous fluid, PBMCs are considered to be an ideal diagnostic material for early DR due to their easy accessibility (22). Our study focused on finding the immune-associated diagnostic genes as biomarkers of early DR and, thus, providing more valuable clues to the pathological mechanism of DR occurrence.

Through our research, three diagnostic genes, namely, *FAM209B*, *POM121L1P*, and *PTGES*, were firstly obtained, and they all showed increased expression levels in DR individuals compared to DM individuals. The discovery of a correlation between *POM121L1P* and type 2 diabetes mellitus (T2DM) has been reported (23). *PTGES* is the terminal enzyme in the biosynthetic process of prostaglandin E2 (PGE2); *PTGES* could also respond to the stimulation brought by inflammation through a method of catalyzing the conversion of prostaglandin endoperoxide H2 (PGH2) to PGE2, and act as a core regulator in inflammation response (24), fever (25), and pain (26, 27). In addition, *PTGES* has also been found to be associated with T2DM. A literature reported that the characteristics of β-cell were better, the aging degree of β-cell was less, the insulin secretion in response to glucose was enhanced, and the glucose steady state was improved in systemic microsomal PTGES-2-deficient mice fed a fat-rich diet or bred with *db/db* mice, a hereditary model of T2DM. Furthermore, they concluded that microsomal PTGES-2 promotes aging of β-cells and their hypofunction via the PGE/EP3/NR4A1 axis, and drug blocking of microsomal PTGES-2 might have a therapeutic effect on aging-induced beta cellular hypofunction and diabetes (28). Also, *PTGES* has also been reported to play a vital role in other autoimmune diseases, including ulcerative colitis (29) and rheumatoid arthritis (30). In addition, the diagnostic model constructed by these three key genes has a good diagnostic efficiency and could distinguish DR from DM effectively, which means that it is possible for us to evaluate and predict the occurrence of DR based on the expression differences of key genes in different diabetic individuals or the changes in the expression of crucial genes at different periods in the same diabetic individual, so that we could hopefully take intervention measures in the early preclinical stage of DR instead of just waiting for the occurrence of DR before taking measures.

Through GSEA, we found that these three diagnostic genes all have correlation with immune-related response. *FAM209B* significantly enriched the functions of regulation of inflammatory response, the pathway of antigen processing, and presentation, while the humoral immune response, immunoglobulin complex, and the pathways of some autoimmune diseases like Alzheimer’s disease and Parkinson’s disease were highly enriched by *POM121L1P* and *PTGES*. A literature reported that the microsomal PTGES-1/PGE axis promotes the process of wound repair by gathering regulatory T cells (31). Another study revealed that targeting the IL-17/microsomal PGES-1/PPAR-γ axis might be a possible approach for the treatment of inflammatory and immune-mediated diseases (32). In addition, it has been found that microsomal PTGES-1 in cells derived from bone marrow might play a crucial role in contact hypersensitivity. PTGES-1-derived *PGE* might promote acquired cutaneous immune responses (33). Other research has shown that exogenous nicotinamide adenine dinucleotide promotes the expression of PTGES and maintains the integrity of the mucus layer that modulates immune response appropriately and therefore participates in the pathological process of inflammatory bowel diseases (34). However, up to now, there was no definitive literature reporting the role of these three diagnostic genes in ocular immune-related inflammatory reactions. Therefore, our study explored the diagnostic value of these three immune-associated genes in distinguishing DR from DM for the first time, as well as their potential key roles in DR triggering mechanisms. According to our findings, there may be a close relationship between these three key diagnostic genes. The single-gene GSEA results of three diagnostic genes are all enriched in immune-related items such as immune regulation, antigen processing, and presentation. Therefore, from the perspective of function or pathway relationships, these diagnostic genes may participate in similar biological pathways. Secondly, from the point of view of the complementary relationship between the three genes in diagnosis, the nomogram constructed by integrating the expression of key genes and the good prediction performance of the three genes as a whole model proved that the three genes played a complementary role in DR diagnosis. However, owing to the lack of direct correlation research literature on these genes by now, further experiments and functional analysis are needed by collecting more clinical samples in the future to gain a deeper understanding of the functions, regulation, and interactions between these diagnostic genes in DR.

Through KEGG, the signaling pathway of antigen processing and presentation enriched by *FAM209B* and the pathway of oxidative phosphorylation co-enriched by *POM121L1P* and *PTGES* have attracted our attention. These deregulated biological processes and pathways are directly or indirectly related to the pathological process and symptoms of DR. The role of inflammatory response in the pathogenesis of DR has been widely confirmed, and retinal inflammation can be detected from early DR to late DR that endangers vision. Abnormal activation of inflammatory response triggers a series of retinal cellular dysfunction and tissue damage (35, 36). The main mechanisms of inflammatory response in DR are leukocyte stasis, infiltration of innate immune cells (such as macrophages and neutrophils) and adaptive immune cells (such as B and T cells), activation of microglia, complement coagulation cascade, upregulation of cytokines, and increment of chemokine composition (37–39). Subsequently, leukostasis can cause retinal microvascular occlusion, and the increased pro-inflammatory mediators, adhesion molecules, chemokines, and growth factors can cause damage to the blood–retinal barrier, and subsequently, capillary leakage can cause macular edema and retinal pathological neovascularization, which lead to decreased vision (40). Meanwhile, the activation of retinal inflammation in DR is inseparable from the antigen processing and presentation pathway in the immune process. In the state of diabetes, extracellular advanced-glycation end products mediate strong pro-inflammatory effects by binding to and activating their receptor, as well as Toll-like receptor 4 on the professional antigen-presenting cells (including monocytes, dendritic cells, and macrophages) (41) expressing major histocompatibility complex class II (MHC II) molecule (42, 43). In addition, a study on diabetes *Ob/Ob* mice also found that the change of MHCII antigen presentation may promote the occurrence of complications of type 2 diabetes (44). The disorder of humoral immune response is also involved in the occurrence and development of DR. Recently, it has been shown that levels of circulating oxidized low-density lipoprotein immune complexes (ox-LDL-ICs) predict the development of DR. Moreover, ox-LDL-ICs exist in the retina of people with type 2 diabetes and is proportional to the severity of DR, which may be related to the cytotoxicity of ox-LDL-ICs toward retinal pericytes (45). A previous study found that the use of regulatory peptide imunofan can normalize humoral immunity and correct immune dysfunction in patients with type 2 diabetic foot syndrome (46). Oxidative phosphorylation (OxPhos) is involved in the maintenance of glucose homeostasis (47). Chronic hyperglycemia leads to a decrease in OxPhos, followed by excessive production of reactive oxygen species (ROS) (48, 49), resulting in oxidative stress-induced damage to the structure and function of retinal microvasculature, including the thickening of capillary basement membrane, the breakdown of blood–retinal barrier, and the formation of acellular and occluded capillaries. Also, both inflammation and angiogenesis are dramatically augmented by hyperglycemia-mediated oxidative stress (50).

As we know, choroidal vessels were more susceptible to changes in immune components of peripheral blood than retina (51). Exposure of RPE/choroid to circulating immune media could affect retinal immune homeostasis (52). A recent work has shown that the fibrovascular membrane in PDR has a different immune landscape compared with that in normal retina (53). Our study firstly compared the differences in proportions of various types of immune cells between early diabetic retinopathy (non-proliferative DR) and DM patients without DR, which is more helpful for understanding the possible triggering mechanism of DR. We found that the proportion of CMPs, iDCs, and naive B cells in the DR group were reduced markedly compared with the DM group, and these three types of immune cells all contributed to the mediation of immune tolerance processes and have been proven to play crucial roles in the pathogenesis of other kinds of autoimmune diseases associated with immune tolerance disorder. CMPs were precursors of monocytes and could differentiate into myeloid cells, including dendritic cells (DCs), macrophages, and granulocyte lineage cells. DCs were the mightiest professional antigen-presenting cells in the body. Among the DCs, iDCs were the main force mediating the process of immune tolerance. The involvement of immune tolerance disorders mediated by iDC reduction in the pathogenesis of systemic autoimmune diseases such as systemic lupus erythematosus, multiple sclerosis, and autoimmune encephalitis has been extensively studied (54), while the research of iDCs in eye diseases mainly focuses on uveitis (55). Naive B cells were developed from immature B cells through mechanisms such as clonal clearance, receptor editing, and inactivation in the bone marrow to form immune tolerance to the body’s own antigens. Therefore, to some extent, naive B cells also reflect the degree of immune tolerance and participate in the immune regulation process. The type 1 DM-derived immune system included reduced proportion of naive B cells compared with healthy controls of personalized immune mice, and similar changes have been observed in systemic lupus erythematosus individuals (56). We speculate that when iDCs and naive B cells are reduced, the immune tolerance at the local choroid–retinal interface is out of balance, and the original immune homeostasis cannot be maintained, thus activating and amplifying the immune inflammatory reaction at the choroid–retinal interface, causing damage to the outer blood–retinal barrier, and then causing excessive autoimmune inflammatory reaction in the retina, thus triggering early DR. However, this hypothesis still needs to be verified by future multi-dimensional experiments.

Furthermore, we integrated 76 TFs, 59 miRNAs, and 2 diagnostic genes to build the TF–mRNA–miRNA regulatory network. From the TF–mRNA–miRNA regulatory network we predicted, it can be seen that there are some common transcription factors (such as ESR1) and miRNAs (including hsa-miR-671-5p, hsa-miR-939-5p, hsa-miR-6752-5p, hsa-miR-6858-5p, hsa-miR-6824-5p, hsa-miR-6765-5p, and hsa-miR-6089) between *FAM209B* and *PTGES.* Among them, *ESR1* was a hub gene in many autoimmune diseases (57), including type 1 diabetes (58), systemic lupus erythematosus (59), and rheumatoid arthritis (60). *ESR1* was widely expressed in thymocytes, T cells, B lymphocytes, etc., which serve roles in the functioning of the immune system and reducing inflammation. Recent research has found that Runx1 indirectly led to reduced expression of trefoil factor family 1 by the CBF-β/*ESR1* axis in mouse retinal microvascular endothelial cells treated with high glucose, which was involved in the occurrence of DR (61). Among the common miRNAs, miR-671-5p has been discovered to be involved in inflammatory and immunomodulation (62) processes in many kinds of diseases, such as periodontitis (63), pulmonary inflammatory injury (64), Parkinson’s disease (65), and atherosclerosis (66). It has been found that miR-939-5p has an anti-inflammatory effect in human aortic endothelial cells (67), and it also modified the apoptosis of endothelial and myocardial cells induced by inflammatory cytokines (68). MiR-6752-5p was associated with cerebrovascular disorder (69). MiR-6858-5p was associated with growth, invasion, and angiogenesis of glioblastoma multiforme (70), and miR-6765-5p was associated with wound healing (71). As for miR-6089, it has been reported to have correlation with the risk of ischemic stroke (72) and rheumatoid arthritis (73). Although these core TFs and miRNAs in this regulatory network have not been reported in T2DM or DR, most of them have been reported to play crucial inflammatory and immunomodulatory roles in other chronic inflammation-related diseases, which has certain similarity and correlation with our findings.

Despite the application of comprehensive bioinformatics analysis methods and RNA expression validation of key genes, our research still has certain limitations. First of all, in DM patients, the expression pattern of immune cell proportions in different tissues and organs may be different. Although it is difficult to obtain retinal tissue samples from DM patients with or without DR, further studying it will be worth the effort, and the difference in the expression of immune-related biomarkers targeting different immune cells needs to be further verified by combining single-cell atlas and immune cell-related experiments in the future; in addition, other large sample size datasets with consistent sample types and degree of disease progression need to be analyzed to validate the gene diagnostic performance and expression trends we observed in the self-sequencing data. Secondly, the design type of this study belongs to a case–control study, and it is necessary to conduct prospective cohort studies with a large sample size in the future to accurately verify the causal relationship between the expression trends of key genes observed in our self-sequencing data and the progression of the disease, as well as its clinical practical diagnostic efficacy. Thirdly, further *in vivo* and *in vitro* experiments are needed in the future to verify the specific pathological and molecular mechanisms of these three immune-associated diagnostic genes in the occurrence of DR.

In conclusion, this study identified three immune-associated diagnostic genes, namely, *FAM209B*, *POM121L1P*, and *PTGES*, as biomarkers associated with immune scores in DR for the first time, and the changes in immune cell landscape in PBMCs may be related to these biomarkers. This finding might be a catalyst for the exploration of the DR triggering mechanism in a T2DM population, and might help to understand the role of immune-associated genes in the molecular mechanism of DR occurrence more deeply.

## Data availability statement

The original contributions presented in the study are publicly available. This data can be found here: https://www.ncbi.nlm.nih.gov/sra/PRJNA975053.

## Ethics statement

The studies involving humans were approved by the Second Affiliated Hospital of Xi’an Jiaotong University ethical review committee. The studies were conducted in accordance with the local legislation and institutional requirements. The participants provided their written informed consent to participate in this study.

## Author contributions

Data analysis and writing: YZh. Editing and providing comments: WZ. Review and revision: JW. Conceptualization and methodology: YZu. All authors contributed to the article and approved the submitted version.
