# Supplementary material for: Identification of biomarkers associated with immune scores in diabetic retinopathy
Source: Front Endocrinol (Lausanne). 2023 Oct 5;14:1228843. doi: 10.3389/fendo.2023.1228843 (PMC10585271; doi:10.3389/fendo.2023.1228843)
Supplement: Supplementary file 1 [file DataSheet_1.zip › Supplementary Material/Supplementary Material.DOCX]

Supplementary Material

# Supplementary Data

The datasets generated for this study can be found in the Sequence Read Archive (SRA), and the accession number in SRA is PRJNA975053. The data can be found here: https://www.ncbi.nlm.nih.gov/sra/PRJNA975053

# Supplementary Tables

**Supplement Table 1**. 456 immune-related genes obtained from ImmPort database.

**Supplement Table 2**. Quantitative real-time PCR primers used in the study.

**Supplement Table 3**. Data statistics of transcriptome sequencing after quality control.

**Supplement Table 4**. Notes on the GO function of differential expressed genes between DR group and DM group.

**Supplement Table 5**. Notes on the KEGG pathway of differential expressed genes between DR group and DM group.

1. **Supplementary Figures**

**
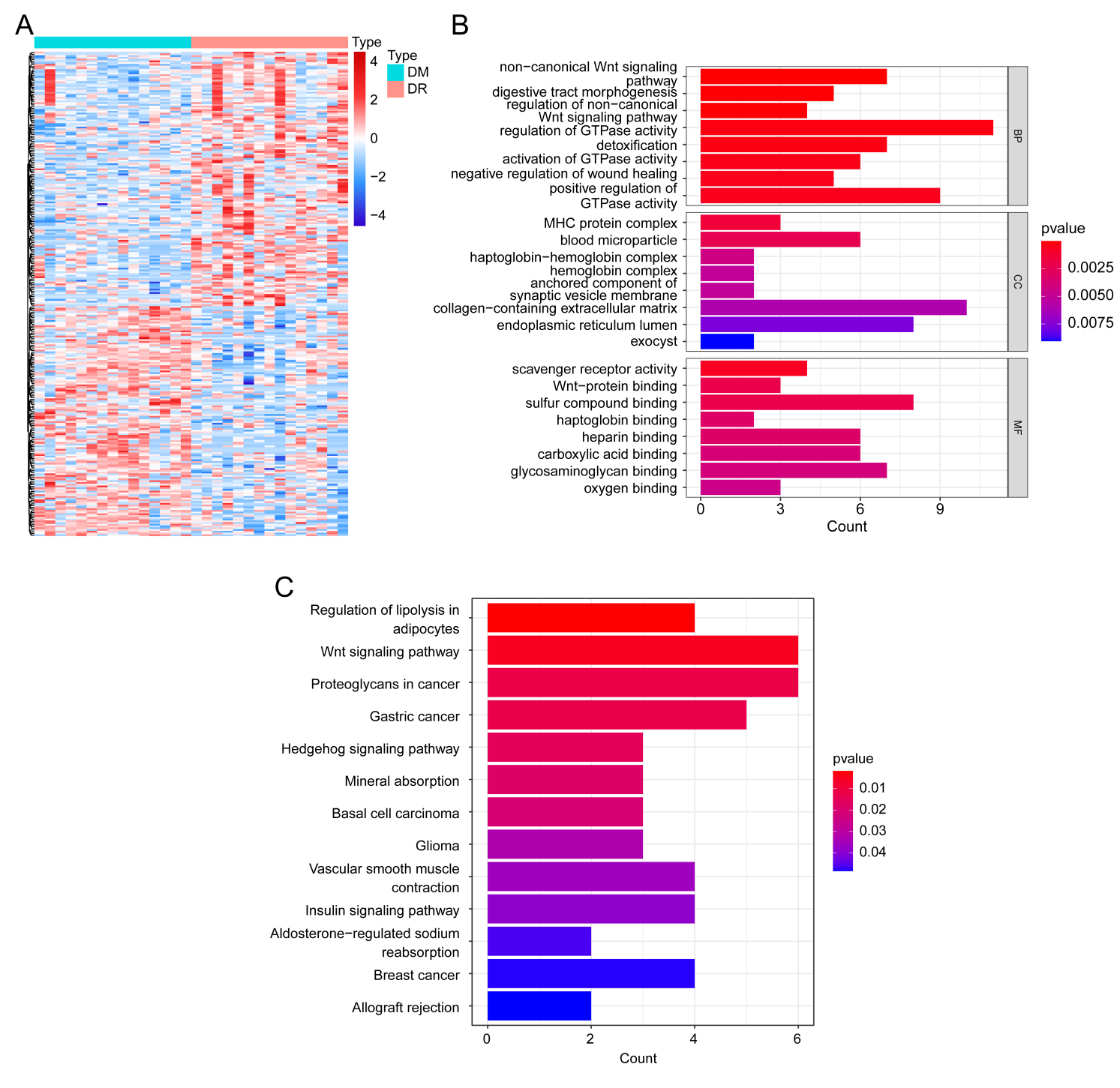
**

**Supplementary Figure 1.** Analysis of the immune-associated differential expressed genes between DR group and DM group. **(A)** Heatmap of differential expressed genes between DR group and DM group, with low expression in blue and high expression in red. **(B)** Enrichment results of differential expressed genes between DR group and DM group by GO analysis. **(C)** Enrichment results of differential expressed genes between DR group and DM group by KEGG analysis.


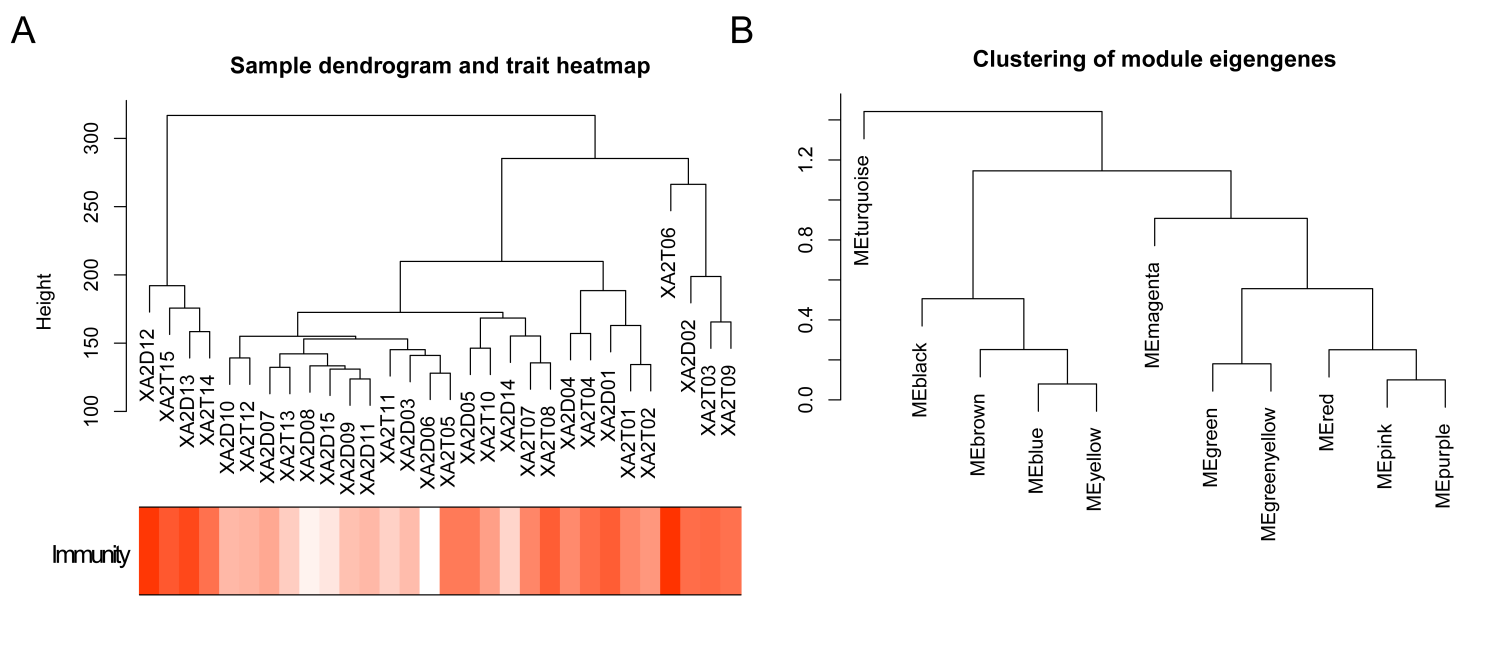


**Supplementary Figure 2.** Sample and module dendrograms. **(A)** Sample dendrogram and trait heatmap. **(B)** Clustering of 11 modules based on similar gene expression patterns.
