# Supplementary material for: Identification of biomarkers associated with immune scores in diabetic retinopathy
Source: Front Endocrinol (Lausanne). 2023 Oct 5;14:1228843. doi: 10.3389/fendo.2023.1228843 (PMC10585271; doi:10.3389/fendo.2023.1228843)
Supplement: Supplementary file 1 [file DataSheet_1.zip › Supplementary Material/Supplementary Table 2. Quantitative real-time PCR primers used in the study..docx]

**Supplement Table 2**. Quantitative real-time PCR primers used in the study.

| Gene | Primer Sequence (from 5' to 3') | |
| --- | --- | --- |
| FAM209B F | CTTCCCATTTCGCACTCCAC | |
| FAM209B R | CAAATTTCAAAAGCTCCACTTCAA | |
| PTGES F | CCCAAGGTTTGAGTCCCTCC |  |
| PTGES R | CACATCTCAGGTCACGGGTC |  |
| POM121L1P F | CCCCACGTTGGGGTCACTA |  |
| POM121L1P R | CCCTGGGTCTGTGTTTGAGGA |  |
| GAPDH F | CGAAGGTGGAGTCAACGGATTT |  |
| GAPDH R | ATGGGTGGAATCATATTGGAAC |  |
