# Supplementary material for: Identification of biomarkers associated with immune scores in diabetic retinopathy
Source: Front Endocrinol (Lausanne). 2023 Oct 5;14:1228843. doi: 10.3389/fendo.2023.1228843 (PMC10585271; doi:10.3389/fendo.2023.1228843)
Supplement: Supplementary file 1 [file DataSheet_1.zip › Supplementary Material/Supplementary Table 3. Data statistics of transcriptome sequencing after quality control..docx]

**Supplement Table 3. Data statistics of transcriptome sequencing after quality control**

| **Sample** | **Total Reads** | **QC%** | **Total Mapped** | **Ratio** |
| --- | --- | --- | --- | --- |
| XA2T01 | 52346046 | 49 | 43308606 | 82.74% |
| XA2T02 | 37918772 | 51 | 34408850 | 90.74% |
| XA2T03 | 40163328 | 50 | 37718165 | 93.91% |
| XA2T04 | 42845140 | 51 | 40478084 | 94.48% |
| XA2T05 | 44222240 | 50 | 41041965 | 92.81% |
| XA2T06 | 47721100 | 50 | 44843214 | 93.97% |
| XA2T07 | 54909930 | 50 | 51933474 | 94.58% |
| XA2T08 | 46933528 | 50 | 44115461 | 94.00% |
| XA2T09 | 45398290 | 50 | 42697813 | 94.05% |
| XA2T10 | 57878018 | 49 | 54826364 | 94.73% |
| XA2T11 | 71537506 | 50 | 66729499 | 93.28% |
| XA2T12 | 41437154 | 55 | 38775275 | 93.58% |
| XA2T13 | 56210984 | 54 | 53187714 | 94.62% |
| XA2T14 | 68929718 | 51 | 64679335 | 93.83% |
| XA2T15 | 54614486 | 49 | 51474569 | 94.25% |
| XA2D01 | 56591382 | 50 | 53376519 | 94.32% |
| XA2D02 | 56210370 | 50 | 53249251 | 94.73% |
| XA2D03 | 53434036 | 51 | 50237246 | 94.02% |
| XA2D04 | 56595886 | 51 | 53695032 | 94.87% |
| XA2D05 | 59434820 | 51 | 56823913 | 95.61% |
| XA2D06 | 54478854 | 55 | 50755889 | 93.17% |
| XA2D07 | 44315734 | 51 | 42028785 | 94.84% |
| XA2D08 | 47728124 | 50 | 45341745 | 95.00% |
| XA2D09 | 44459312 | 51 | 40343125 | 90.74% |
| XA2D10 | 48643928 | 49 | 46280239 | 95.14% |
| XA2D11 | 46998328 | 50 | 44708813 | 95.13% |
| XA2D12 | 47187346 | 49 | 44730676 | 94.79% |
| XA2D13 | 48788856 | 50 | 46423646 | 95.15% |
| XA2D14 | 46301562 | 55 | 43692613 | 94.37% |
| XA2D15 | 45772586 | 55 | 43628880 | 95.32% |
